# Asynchronous axonal firing patterns evoked via continuous subthreshold kilohertz stimulation

**DOI:** 10.1088/1741-2552/acc20f

**Published:** 2023-03-16

**Authors:** Luis Vargas, Eric D Musselman, Warren M Grill, Xiaogang Hu

**Affiliations:** 1 Joint Department of Biomedical Engineering, University of North Carolina at Chapel Hill and North Carolina State University, Chapel Hill, NC, United States of America; 2 Department of Biomedical Engineering, Duke University, Durham, NC, United States of America; 3 Department of Electrical and Computer Engineering, Duke University, Durham, NC, United States of America; 4 Department of Neurobiology, Duke University, Durham, NC, United States of America; 5 Department of Neurosurgery, Duke University, Durham, NC, United States of America; 6 Department of Mechanical Engineering, Pennsylvania State University, University Park, PA, United States of America; 7 Department of Kinesiology, Pennsylvania State University, University Park, PA, United States of America; 8 Department of Physical Medicine & Rehabilitation, Pennsylvania State Hershey College of Medicine, Hershey, PA, United States of America; 9 Huck Institutes of the Life Sciences, Pennsylvania State University, University Park, PA, United States of America; 10 Center for Neural Engineering, Pennsylvania State University, University Park, PA, United States of America

**Keywords:** transcutaneous nerve stimulation, axon activation, kilohertz stimulation, asynchronous axon firing

## Abstract

**Objective.:**

Transcutaneous electrical stimulation of peripheral nerves is a common technique to assist or rehabilitate impaired muscle activation. However, conventional stimulation paradigms activate nerve fibers synchronously with action potentials time-locked with stimulation pulses. Such synchronous activation limits fine control of muscle force due to synchronized force twitches. Accordingly, we developed a subthreshold high-frequency stimulation waveform with the goal of activating axons asynchronously.

**Approach.:**

We evaluated our waveform experimentally and through model simulations. During the experiment, we delivered continuous subthreshold pulses at frequencies of 16.67, 12.5, or 10 kHz transcutaneously to the median and ulnar nerves. We obtained high-density electromyographic (EMG) signals and fingertip forces to quantify the axonal activation patterns. We used a conventional 30 Hz stimulation waveform and the associated voluntary muscle activation for comparison. We modeled stimulation of biophysically realistic myelinated mammalian axons using a simplified volume conductor model to solve for extracellular electric potentials. We compared the firing properties under kHz and conventional 30 Hz stimulation.

**Main results.:**

EMG activity evoked by kHz stimulation showed high entropy values similar to voluntary EMG activity, indicating asynchronous axon firing activity. In contrast, we observed low entropy values in EMG evoked by conventional 30 Hz stimulation. The muscle forces evoked by kHz stimulation also showed more stable force profiles across repeated trials compared with 30 Hz stimulation. Our simulation results provide direct evidence of asynchronous firing patterns across a population of axons in response to kHz frequency stimulation, while 30 Hz stimulation elicited synchronized time-locked responses across the population.

**Significance.:**

We demonstrate that the continuous subthreshold high-frequency stimulation waveform can elicit asynchronous axon firing patterns, which can lead to finer control of muscle forces.

## 1. Introduction

ranscutaneous electrical stimulation of the nervous system is used to strengthen impaired muscles through repetitive stimulation and as an assistive strategy to reanimate impaired or paralyzed muscles [1–4]. Stimulation electrodes are typically placed on the skin over the muscle belly near the motor point to activate the distal ends of innervating nerve fibers [5]. Alternatively, electrodes can be placed around major peripheral nerve bundles to activate a range of muscles [6]. The stimulation intensity is adjusted to control the level of muscle activation (recruitment), and this technique is easy to implement in clinical and home settings for basic gross movements involving low degree-of-freedom control (such as hand opening and closing or ankle dorsiflexion). However, several issues limit clinical adoption of functional electrical stimulation (FES). For example, each stimulation pulse can evoke a single action potential in the active fibers, which leads to highly synchronous activity across the activated population [7]. In contrast, during voluntary contractions asynchronous firing of individual motor units occurs. Highly synchronized axonal activation leads to onset of muscle fatigue rapidly and increased force variability (i.e. highly synchronized force twitches lead to rapid force variations) [8–11], thereby limiting clinical utility with conventional stimulation waveforms. Herein, we describe experimental and modeling studies of kHz frequency electrical stimulation to generate asynchronous activation of motor units.

Both spatial and temporal parameters have been explored to generate asynchronous muscle activation. For example, multi-channel stimulation was used to target different muscle regions using specific groups of contacts [12–14]. By alternating which electrodes delivered current, distinct muscle regions were activated asynchronously, thereby delaying muscle fatigue. Similarly, electrode arrays (either implantable electrodes or surface electrodes) placed near the nerve bundle generate distinct electrical fields when current is delivered to different electrodes, which can activate different sets of nerve fibers and their corresponding muscle regions [15–17]. Although effective in reducing muscle fatigue, a particular channel in the multi-channel array still recruits a set of nerve fibers synchronously, with distinct compound action potential shapes that typically have large amplitudes and well-defined phases [17, 18], due to the sum of many synchronous action potentials from multiple muscle fibers.

The temporal pattern of stimulation can also influence the pattern of axonal recruitment. Bursts of high-frequency stimulation pulses at kHz frequencies can lead to transient changes in axonal firing patterns, including asynchronous firing [19] or conduction block [20]. With amplitude-modulated bursts of stimulation or tonic stimulation at kHz frequencies, different axons fire synchronously initially and then transition to more random asynchronous firing patterns due to different axonal response properties [21]. Although these patterns of asynchronous firing may be beneficial, the initial transient activation can generate undesirable muscle force fluctuations.

The current study presents a novel stimulation waveform with short-duration current pulses delivered continuously (100% duty cycle) at kHz frequency. By varying the current amplitude and pulse width, each short duration pulse evokes subthreshold depolarization of the axonal membrane potential, and the temporal summation of a series of depolarizations evokes an action potential. We hypothesized that axons with different diameters would require different numbers of summed depolarizations, which would lead to asynchronous action potentials, and subthreshold stimulation may prevent the initial transient synchronized responses across axons. We evaluated our stimulation waveform using both model simulations and experimental measurements. Specifically, we modeled extracellular stimulation of mammalian myelinated axons using a simplified volume conductor model to solve for extracellular electric potentials. We modeled axonal firing patterns under this novel kHz stimulation waveform and conventional 30 Hz stimulation (i.e. rectangular biphasic charge-balanced pulses). The simulation was intended to establish the temporal properties of axonal firing patterns, and a simplified volume conductor model, rather than a subject-specific realistic model, reduced the computational complexity and still provided essential information on the axonal firing patterns. Experimentally we recorded muscle contraction output (i.e. electromyogram (EMG) and force) while delivering either the novel waveform or conventional 30 Hz stimulation, or through voluntary muscle contractions. We quantified EMG temporal properties (entropy) and force variability as indirect measures of axonal firing patterns. We demonstrate a novel electrical stimulation waveform that elicits asynchronous firing patterns in peripheral nerve fibers, and this may lead to better control of muscle force and delayed onset of muscle fatigue.

## 2. Materials and methods

### 2.1. Experimental study

#### 2.1.1. Participants

Nine neurologically intact participants (seven males and two females, 22–38 years of age) were recruited to this study. Each participant gave informed consent for the protocols approved by the Institutional Review Board at the University of North Carolina at Chapel Hill.

#### 2.1.2. Experimental setup

**Figure 1.**
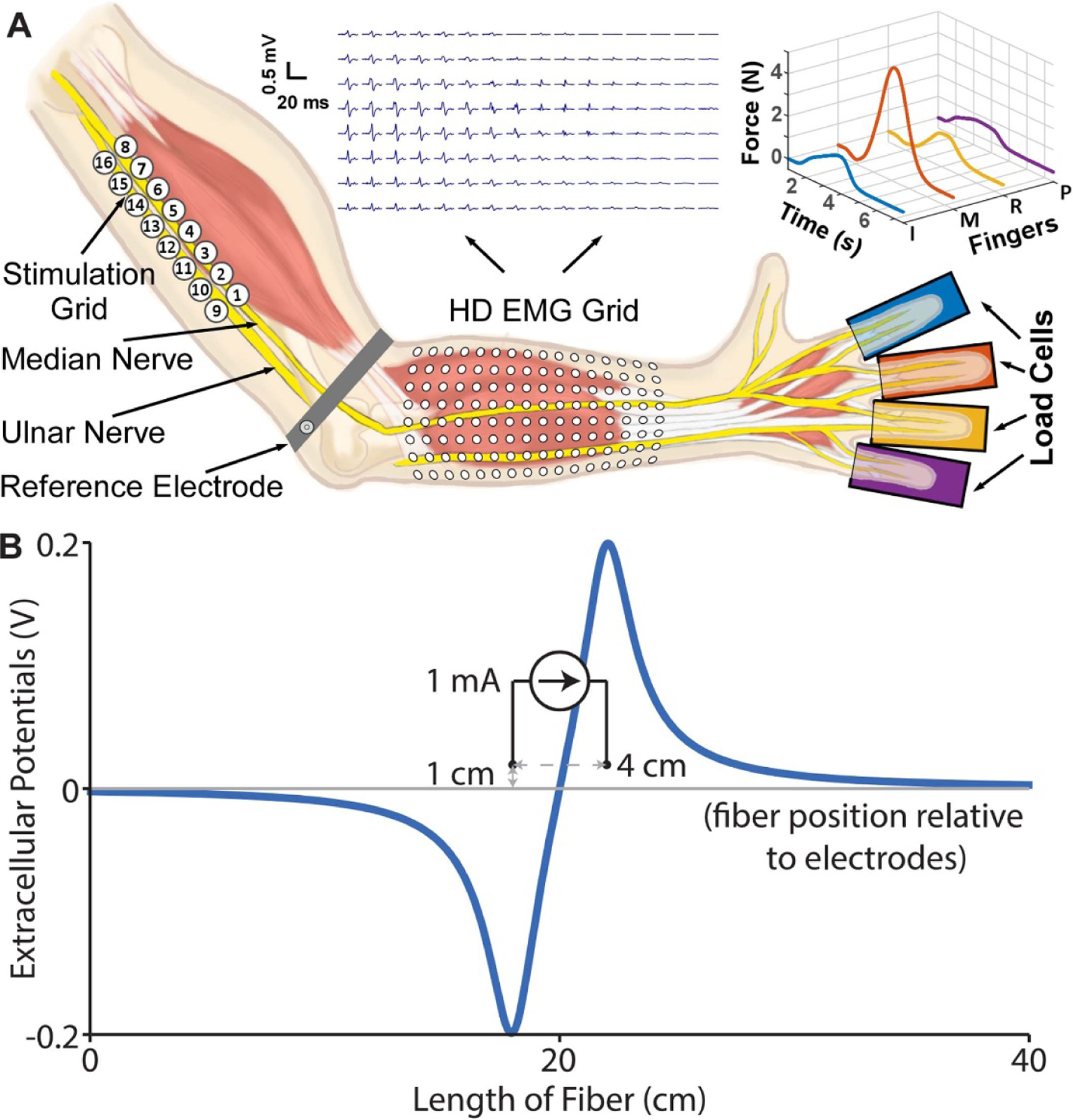
Overview of experimental setup and model diagram. (A) We used a grid of stimulation electrodes along the ulnar and median nerves to activate targeted muscles. We recorded the evoked motor activity using individual load cells and high-density EMG (HD EMG) grid electrodes. Example compound action potentials and finger flexion forces from a conventional low-frequency pulse are shown. (B) Diagram of the computational model, which includes two current sources in bipolar configuration (4 cm pitch, 1 cm from fibers) delivering current controlled stimulation in an infinite, isotropic, homogenous medium representing the tissue surrounding the fiber. The electric potentials from delivering 1 mA are applied extracellularly to the model fibers as a function of time by multiplying them by the stimulation waveform, *I*(*t*).

Participants sat in a height-adjustable chair and were asked to place their forearm on a foam pad on a table in front of them. We cleaned the skin surface along the anterior forearm and medial upper arm with alcohol pads to improve the electrical connection between the stimulation grid and recording electrode array placed on the skin of these two regions, respectively. The stimulation grid had 16 channels (2 × 8) with 1 cm diameter gel-based electrodes. We placed the grid along the medial portion of the upper arm directly below the short head of the biceps brachii (figure 1). Under this skin surface, the median and ulnar nerve bundles run approximately along a vector connecting the center of the axilla and the medial epicondyle of the humerus. We selected this location because the targeted nerve bundles run closely in parallel and are near the skin surface. Previous work has shown that a variety of hand grasp patterns can be produced with bipolar stimulation to activate functionally distinct groups of axons [6, 22].

To record isometric finger flexion forces, we positioned the participant’s hand between two stiff foam pads located along the palmar and dorsal sides of the hand. The participants’ four fingers (i.e. index, middle, ring, and pinky) were abducted comfortably and placed on four miniature load cells (SM-200N, Interface, Scottsdale, AZ). We adjusted the orientation of each load cell to match the orientation of participants’ respective fingers to ensure comfort and accurate recording of the flexion forces. We used Velcro straps to secure each finger to the load cell. We asked participants to report any discomfort or if the Velcro straps loosened at any point during the experiment. We sampled the signal from the load cells at 1 kHz. We also recorded the activation patterns of the finger flexor muscles using a high-density (16 × 8) electromyogram (HD EMG) electrode array placed on the anterior surface of the forearm (figure 1(A)). The placement location of the electrode array was determined through palpitation of the muscle belly as participants actively flexed their individual fingers such that the electrode array covered a majority of the flexor muscles. Each electrode of the HD EMG array was 3 mm in diameter and separated with an inter-electrode spacing of 10 mm (center-to-center). We acquired monopolar HD EMG recordings using the EMG-USB2+ system (OT Bioelettronica, Torino, Italy) and a reference electrode placed along the individual’s upper arm just proximal to the elbow. The EMG signals were sampled at a frequency of 2048 Hz, amplified by a gain of 200, and bandpass filtered at 10–900 Hz.

**Figure 2.**
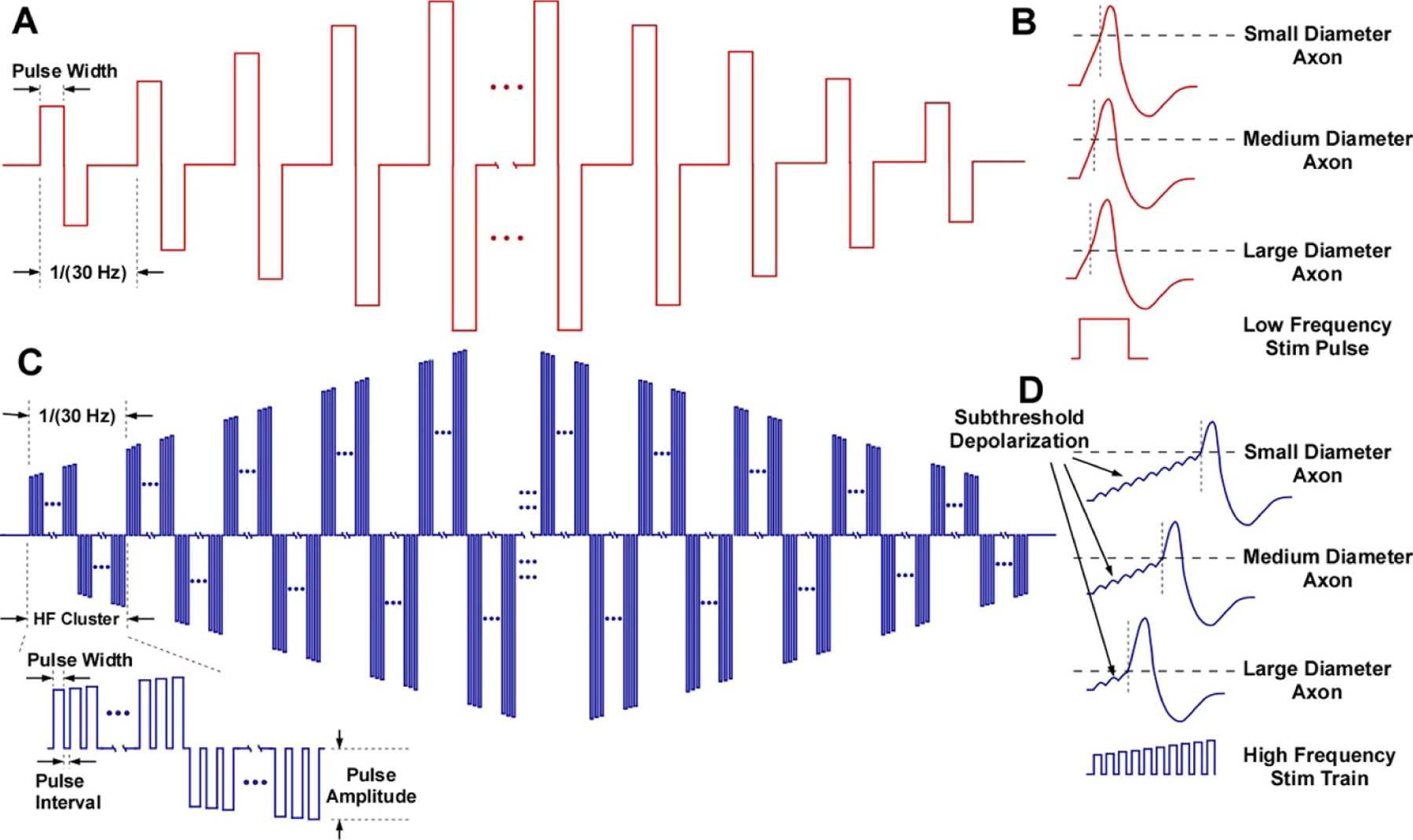
Stimulation waveforms and hypothesized fiber responses. (A) Conventional low frequency (LF) stimulation waveform. Charge-balanced pulses are delivered at 30 Hz with pulse amplitudes ramped up over 2 s and then down over 2 s. (B) Individual LF pulse elicits action potentials in axons with different thresholds, with highly synchronized timing of activation. (C) High frequency (HF) stimulation waveform. Clusters (delivered in 30 Hz) contain bursts of short charge-balanced pulses with amplitudes ramped up over 2 s and then down over 2 s. Each cluster (bottom-left zoomed view) contains a series of positive short pulses followed by an equal number of negative pulses with the same sequence of amplitudes as the burst in the positive phase. For example, the first positive pulse and the first negative pulse in each HF cluster has the same amplitude (charge-balanced). (D) A series of subthreshold depolarizing responses evoked by the short HF pulses can sum to trigger an action potential. Axons with different recruitment thresholds require differing degrees of subthreshold polarization to trigger an action potential, thereby causing asynchronous activation.

We used MATLAB (MathWorks, Natick, MA) to deliver a current-controlled stimulation waveform and select electrode pair in the stimulation array. Specifically, MATLAB controlled a multichannel stimulator (STG4008, Multichannel Systems, Reutlingen, Germany) that created charge-balanced biphasic pulses with temporal resolution of 20 *µ*s and a switch matrix (Agilent Technologies, Santa Clara, CA) linked the anode and cathode of the stimulator to the selected electrode pair. The stimulation waveforms delivered for traditional low frequency (LF) and subthreshold kHz high frequency (HF) stimulation are illustrated in figure 2.

During LF stimulation, we delivered a single biphasic rectangular pulse per period (1/frequency) (figure 2(A)). Stimulation frequency and pulse width were 30 Hz and 500 *µ*s, respectively [23], and the stimulation amplitude was adjusted for individual participants as described below. We expected that the LF stimulation pulses initiate action potentials that are time-locked with the stimulation pulses (figure 2(B)).

During subthreshold HF stimulation, we delivered clusters of stimulation each containing bursts of pulses by adjusting the pulse width and pulse interval (figure 2(C)). The HF pulse interval was set to 20 *µ*s (i.e. stimulator temporal resolution) between any adjacent positive or negative pulses, both within and across pulse clusters (bottom left insert of figure 2(C)). Effectively, the individual pulses spanned continuously across the entire stimulation trial duration (i.e. 100% duty cycle). We set the HF pulse width to either 40, 60, or 80 *µ*s, which resulted in pulse repetition frequencies of 16.67, 12.5, or 10 kHz, respectively. For each HF cluster, the three HF settings contained approximately 556, 416, or 333 HF pulses (50% positive and 50% negative), respectively. These three pulse width values were selected to evaluate the different effect of temporal summation on subthreshold depolarization. Based on our preliminary testing, the individual narrow pulses would not trigger recordable action potentials unless bursts of pulses were delivered at kHz frequency, which indicates that individual pulses only evoked subthreshold depolarization of the axonal membrane potential. We hypothesized that axons with different recruitment thresholds would require different numbers of depolarization pulses, thereby resulting in asynchronous activation of fibers (figure 2(D)). We used a rising or falling ramp within each burst of pulses to produce a monotonically increasing or decreasing amplitude across bursts, respectively. However, the current amplitude range within one HF burst is small (∼16.7 *µ*A: the amplitude ramped ∼2 mA over 2 s (table 1) and each burst lasted ∼16.7 ms). Slight modifications to our HF burst waveform will likely produce consistent results (e.g. constant current amplitude within a burst) since the electrodes are located on the skin surface far from the fibers.

For both LF and HF stimulation conditions, we ramped the current amplitude up over 2 s and back down at the same rate. For each condition, the lower and upper bounds of stimulation amplitude were chosen based on the evoked motor response. The lower amplitude was set to ∼0.5 mA below the motor threshold (table 1), which was defined as the minimum stimulation amplitude that evoked visible motor (EMG and force) responses. We ramped the stimulation amplitude using steps of 0.1 mA until motor responses were observed. We repeated the process three times, and the average value across trials was calculated as the motor threshold. The upper bound of the stimulation amplitude was set to a designated maximum current value that produced moderate force levels (20%–25% of maximum voluntary force), with a matched peak force across all the LF and the three HF stimulation conditions. A moderate contraction level was chosen to minimize subject discomfort during experimental trials, and because low finger muscle forces are typically used during most activities of daily living [24].

### 2.2. Experimental procedure

Prior to the main experiment, the maximum voluntary flexion force of each finger was obtained, during which the participants produced their maximum flexion effort against the load cell for 2 s, and the peak force was determined. We then searched through the stimulation grid for a pair that evoked a particular finger flexion with minimal wrist activation. During the grid search, the stimulator was set to deliver 1 s of conventional LF stimulation to assess quickly the viability of the electrode pair (i.e. could evoke force output without inducing painful sensation) and stimulation level. Once we identified a viable pair, we then determined the lower and upper bounds of the stimulation amplitude for each waveform.

The study was broken into four stimulation blocks and a voluntary contraction block. For each stimulation block, a single stimulation waveform (i.e. one LF or any of the three HF conditions) was assessed. The order of the four blocks was randomized across subjects. Prior to the start of each block, 4 s of ramped (up and down) stimulation pulses was delivered to confirm the evoked peak force was captured and to ensure that the stimulation was comfortable for the participants. Once the participant confirmed their comfort, we performed a longer trial consisting of 20 repetitions of the 4 s stimulation train. Each stimulation train was followed by a 2 s rest period. We allowed a 5 min rest period between blocks to minimize muscle fatigue. Lastly, five trials of voluntary (Vol) contractions were performed in which we asked subjects to replicate voluntarily the triangular force profile evoked during the stimulation blocks.

### 2.3. Modeling study

To model the response of a human median nerve to electrical stimulation, we applied extracellular electric potentials as a time-varying waveform to models of biophysically realistic mammalian myelinated fibers [25, 26] using geometry interpolated from the original diameters [27]. The simulation inputs (i.e. extracellular potentials and stimulation waveforms), data analyses, and data visualization were created using Python v3.7 [28], and the nerve fiber simulations were performed using NEURON v7.6 [29].

We computed the extracellular potentials using the analytical solution (equation (1)) which assumes two-point current sources in bipolar configuration (located *r*_1_ and *r*_2_ distances from a point on the fiber) within an infinite, isotropic, and homogenous medium containing the nerve fibers (electrical conductivity: *σ* = 0.3 S m^−1^ to approximate the tissue surrounding the median nerve),

(1) $$\text{Φ}=\frac{I}{4\pi \sigma r_{1}}-\frac{I}{4\pi \sigma r_{2}}.$$

We modeled the electrodes with 4 cm separation (i.e. electrode pitch) similar to the experiment setting, and the fibers were placed 1 cm away and parallel to the electrodes (figure 1(B)); we aligned the central node of Ranvier halfway between the electrodes. We randomly selected diameters from a truncated normal distribution 9 ± 7 *µ*m (mean ± 2*standard deviation (SD)) to capture known variability in *α* motor neuron axon diameter [30]. Across all 100 fibers we modeled, the total length was conserved (i.e. 400 mm), which resulted in varying number of nodes of Ranvier across fiber diameters.

The model axons were initialized to steady state by using long (i.e. 1 ms) timesteps for 20 ms before applying the stimulation waveform. During the simulation, we used a 1 *µ*s timestep and counted action potentials when the transmembrane potential passed −30 mV with a rising edge. Action potential times were detected at 90% of the fiber length, which ensured that the recorded signals were from propagating action potentials rather than from ohmic increases in transmembrane potential beneath the electrode.

We simulated the LF 30 Hz biphasic symmetric rectangular pulse train and the HF burst waveform. For both waveforms, we ramped the waveform amplitude from 2 to 6 mA over 2 s and then back down at the same rate. We simulated a biphasic symmetric rectangular pulse (0.5 ms phase^−1^) at 30 Hz (figure 2(A)). The HF waveform consisted of alternating polarity bursts with 12.5 kHz pulse repetition frequency (figure 2(C)). Each burst delivered 208 pulses in 16.67 ms (i.e. duration of one phase with 60 *µ*s pulses interleaved by 20 *µ*s off time), which yields a burst frequency of 30 Hz. The odd numbered bursts ramped in amplitude (i.e. monotonically increasing or decreasing pulse amplitudes within each burst for the first 2 s and last 2 s, respectively), and the even numbered bursts mirrored the previous burst in amplitude (i.e. negative current) and time (i.e. sequence of pulse amplitudes were the same as the first phase, but in reversed order) (figure 6(A)). The waveform used experimentally did not mirror the sequence of amplitudes in the even numbered bursts.

### 2.4. Data analysis

#### 2.4.1. HD EMG signal analysis

We evaluated muscle recruitment patterns by calculating the predictability or complexity of the time series of the HD EMG signals. Synchronized axonal activation leads to well-defined compound action potentials with distinct peaks, which lead to high predictability or low complexity of the EMG signals as quantified by an entropy measure (sample entropy [31]: the entropy value ranges from 0 to 2.5 with higher values representing greater complexity or low predictability in the signal time series). In contrast, asynchronous axonal activation, as in voluntary contraction, lead to asynchronously distributed action potentials in time and low predictability or high complexity of the HD EMG signals. We expected that there would not be distinct compound action potentials in the EMG generated by HF stimulation because of asynchronous evoked axon activation. Different numbers of pulses were needed for axons with different diameters and positions in the nerve to generate an action potential. As a result, distinct compound action potentials are not evident in response to HF stimulation. To quantify the EMG signals, first we removed the stimulation artifact in the EMG signals. For LF stimulation, we identified the stimulation artifacts using the timing of the stimulation pulses. Centered at each stimulation artifact, we replaced a 5 ms window with random baseline EMG (a data segment without stimulation or identifiable action potentials). For HF stimulation, we used spike triggered averaging [32] to extract the shape of the stimulation artifact across 4 s of the stimulation train. We aligned the EMG signal of each channel from different HF clusters in each trial. We calculated the average response to extract the shape of the stimulation artifact for each EMG channel. For each 4 s HF stimulation train, we time-aligned and magnitude-adjusted the artifact to compare it with the original HD EMG signal (figure S1 in the supplementary material). Specifically, the amplitude and time alignment of the 4 s stimulation duration were adjusted by minimizing the mean-squared-error between the raw EMG (with artifact) and estimated artifact waveforms, which was necessary to account for stimulation artifact with magnitude several orders of magnitude higher than the EMG signals. Finally, we removed the artifact by subtracting it from the corresponding EMG channel. We could not remove all stimulation artifact from the EMG signal since the amplitude of the artifact over 4 s can fluctuate in amplitude or time. To reduce the impact of residual stimulation artifact on EMG signal analysis, we calculated the root mean square (RMS) values of the processed HD EMG signals and then ranked them in descending order of RMS magnitude. The 30 channels with the highest RMS were excluded based on visual inspection of residual stimulation artifacts, and the next 30 channels were then retained for further analysis. We calculated the sample entropy of the processed EMG for the retained 30 channels in each 4 s stimulation train and averaged the entropy values across channels in each waveform. Our sensitivity analysis (figure S2 in the supplementary material) showed similar results when different sets of channels were selected. We performed entropy analysis on the LF, HF, and voluntary EMG data.

#### 2.4.2. Force signal analysis

We processed the force profiles to quantify the spectral power of the force output at the stimulation frequency (30 Hz) and the variability (i.e. standard deviation) of the force trajectory. We hypothesized that synchronized activation of the axons will lead to high power at 30 Hz and force variability across the 4 s stimulation train from responses being time-locked with the LF stimulus. For each HF, LF, and voluntary trial, we used the finger that produced the maximum force that was matched during the experiment. Prior to evaluating the spectral power, we applied a 5 Hz high pass filter to the forces from each 4 s stimulation train. We performed spectral analysis, using a custom written script based on the ‘*fft*’ function in MATLAB with a 6100-sample window, on the resulting high-pass filtered force signal and averaged the spectral power from 29.5 to 30.5 Hz to calculate the power at 30 Hz. To assess the variability of the produced forces across 20 stimulation trains, we calculated the SD of force across 20 stimuli and at the maximum force produced during the stimulation train. When calculating the SD of the force along the time series from 0.25 to 4.5 s of each stimulation train, we used the average forces within a 50 ms moving window with a moving step of 0.25 s.

### 2.5. Statistical analysis

For each outcome metric (i.e. EMG entropy, spectral power at 30 Hz, and variability of force), we assessed the normality of the results using the Shapiro–Wilk test. All metrics were normally distributed across subjects, except for the power of the force at 30 Hz. For this metric, we used the Kruskal–Wallis test using a chi-square distribution to discern differences across the five experimental conditions. When necessary, we used the Mann–Whitney *U* test and a Bonferroni correction to correct for multiple comparisons. We used repeated measures Analysis of Variance (ANOVA) to discern differences in all other metrics across experimental conditions. We performed post hoc paired comparisons using Tukey’s honestly significant difference test. We performed the statistical analyses in SPSS (IBM, Armonk, NY) with an *α* value of 0.05.

## 3. Results

### 3.1. Experimental results

**Figure 3.**
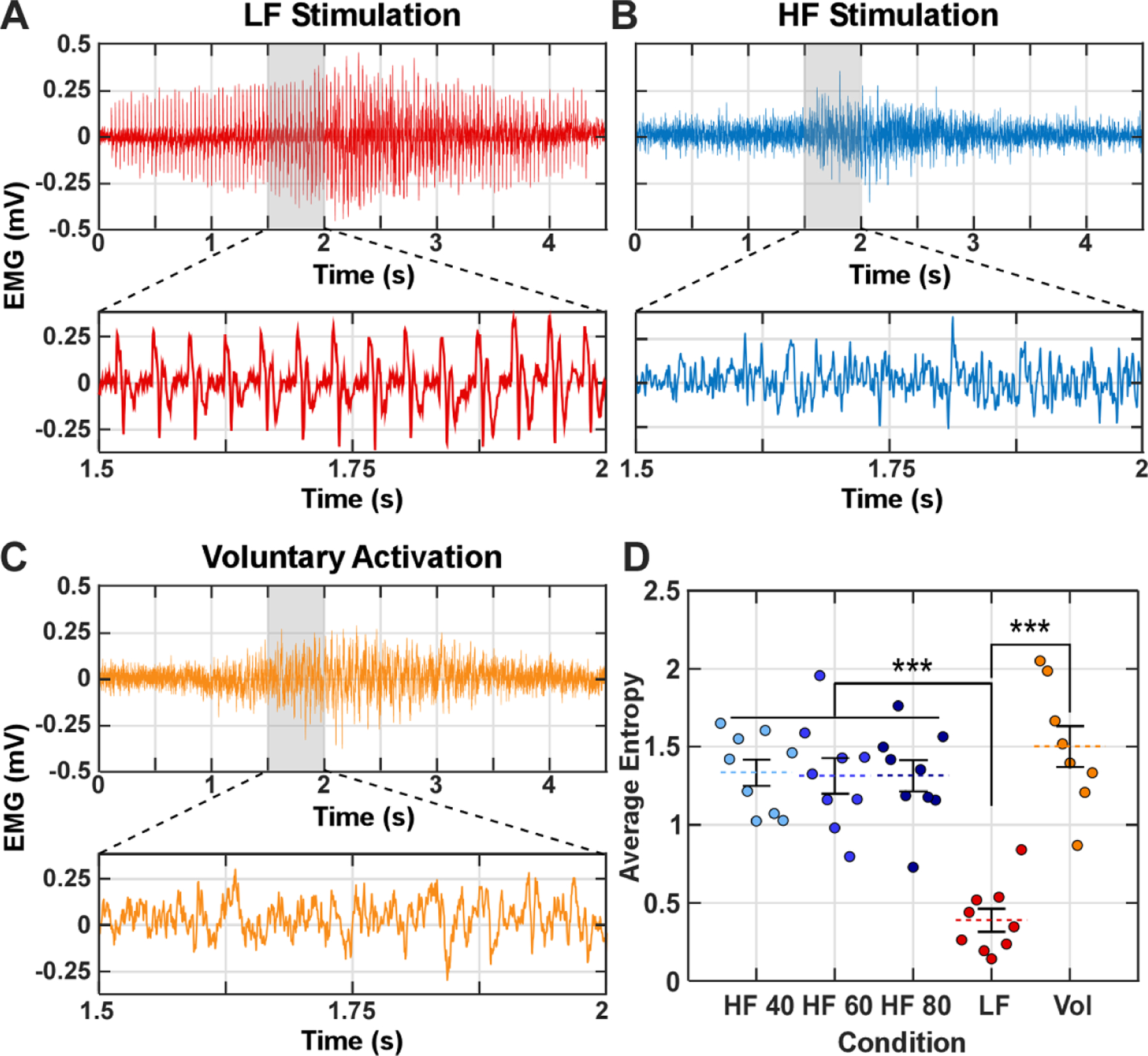
Entropy analysis of EMG response to stimulation compared to voluntary activation (Vol) condition. The time series of EMG for (A) low-frequency (LF) stimulation, (B) high-frequency (HF) stimulation, and (C) during voluntary muscle activation. (D) We assessed three HF conditions labeled ‘HF 40’ (HF with 40 *µ*s pulse width), ‘HF 60’ (HF with 60 *µ*s pulse width), and ‘HF 80’ (HF with 80 *µ*s pulse width). The sample entropy for each condition with error bars indicating standard error across participants. The circles in each condition represent the individual subjects ordered from left to right based on the subject number. We used a one-way ANOVA to evaluate statistical significance (*F* = 18.7, *p <* 0.001) with post hoc paired comparisons conducted using Tukey’s honestly significant difference (HSD) test. *** denotes *p <* 0.001.

We first quantified the temporal dynamics of the EMG to characterize axonal recruitment across stimulation conditions. Examples from a single EMG channel are portrayed for the LF, HF, and voluntary (Vol) conditions in figures 3(A)–(C). LF stimulation resulted in well-defined compound action potentials with distinct peaks (figure 3(A)) from synchronized axonal recruitment. In contrast, HF stimulation led to more random EMG activity (figure 3(B)) that closely resembled the voluntary EMG activity (figure 3(C)). LF stimulation resulted in entropy that was significantly lower than entropy in the HF and Vol conditions (*p <* 0.001, figure 3(D)). Conversely, no significant differences in entropy were detected between the HF and Vol conditions (vol vs HF 40: *p* = 0.55, vol vs HF 60: *p* = 0.63, and Vol vs HF 80: *p* = 0.41).

**Figure 4.**
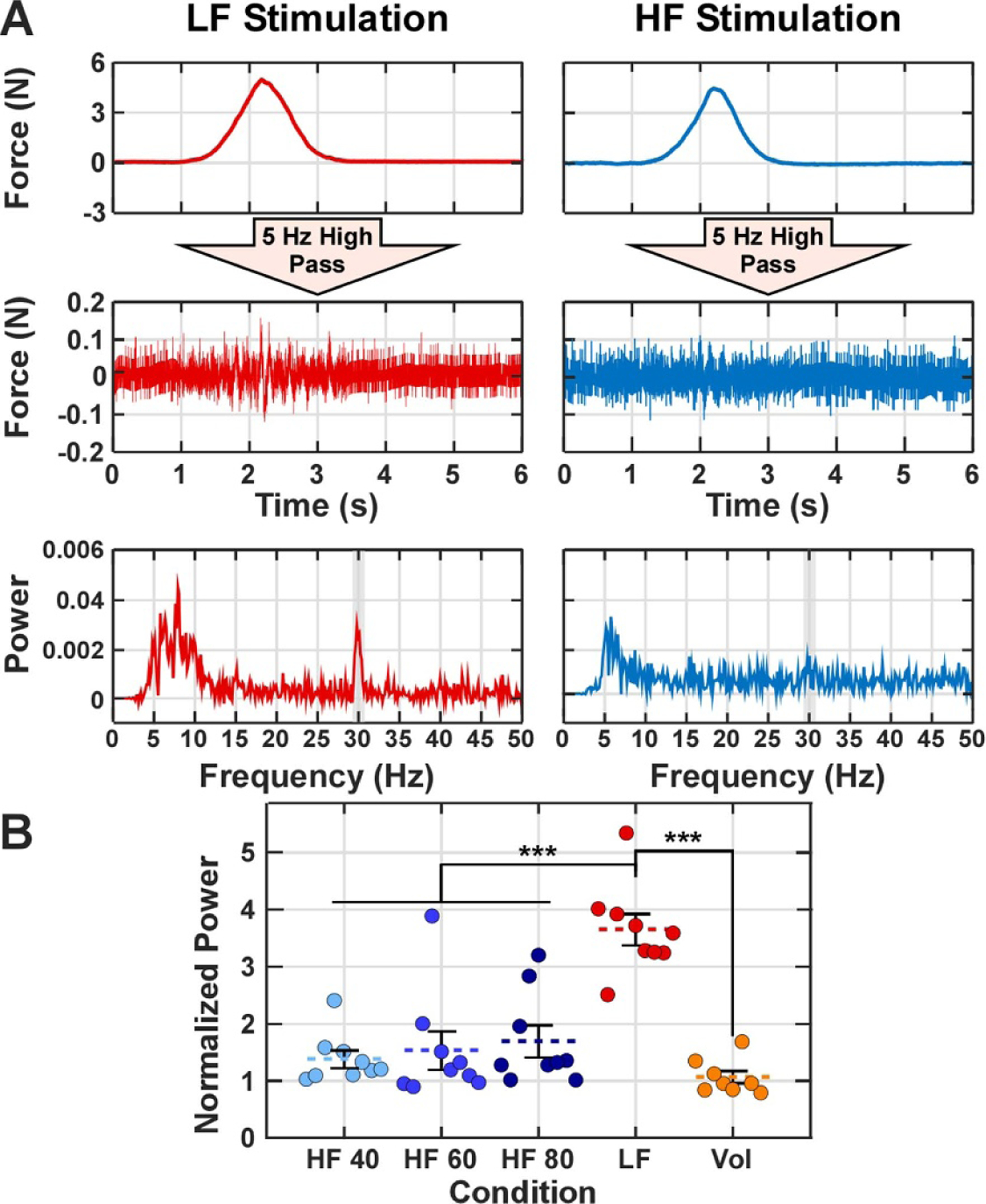
Spectral analysis for force responses to low (LF) stimulation, high (HF) frequency stimulation, and voluntary activation (Vol). (A) Force time series from a 4 s stimulation epoch (top row). High-pass filtered force time series from the same epoch (middle row). Power spectrum of the high-pass filtered force time series (bottom row). The shaded region indicates the power from 29.5 to 30.5 Hz. (B) Normalized power for each experimental condition with the error bars denoting standard error. The circles in each condition represent the individual subjects ordered from left to right based on the subject number. We used a Kruskal–Wallis test to evaluate significance (*H* = 23.4, *p <* 0.001) and a Mann–Whitney *U* test for post hoc paired comparisons. *** denotes *p <* 0.001.

We quantified differences in stimulation-evoked forces across stimulation conditions (HF or LF). We first conducted spectral analysis on the high pass filtered force traces to obtain the power at 30 Hz as a measure of how well muscle force aligned with the carrier frequency of stimulation, with a higher power amplitude signifying greater synchronization of axonal recruitment. Figure 4 depicts the process to obtain the power at 30 Hz for both LF and HF trials and the average normalized power for each stimulation condition across all participants. The normalized power amplitude in the HF conditions was not significantly different from the responses observed during the Vol condition (figure 4(B)). Further, the evoked forces in the LF stimulation had significantly higher spectral power at 30 Hz (*p <* 0.001) than the HF waveform, which suggests robust entrainment of fibers for the LF waveform but not the HF waveform. Subject #4 (the circle outside the main cluster in each condition) showed a consistently higher power at the HF 40, HF 60, and LF conditions compared with other subjects. The amplitude of stimulation current for this subject was indeed higher than other subjects. But subjects #3 and #5 with similar amplitudes of current did not exhibit a high power.

**Figure 5.**
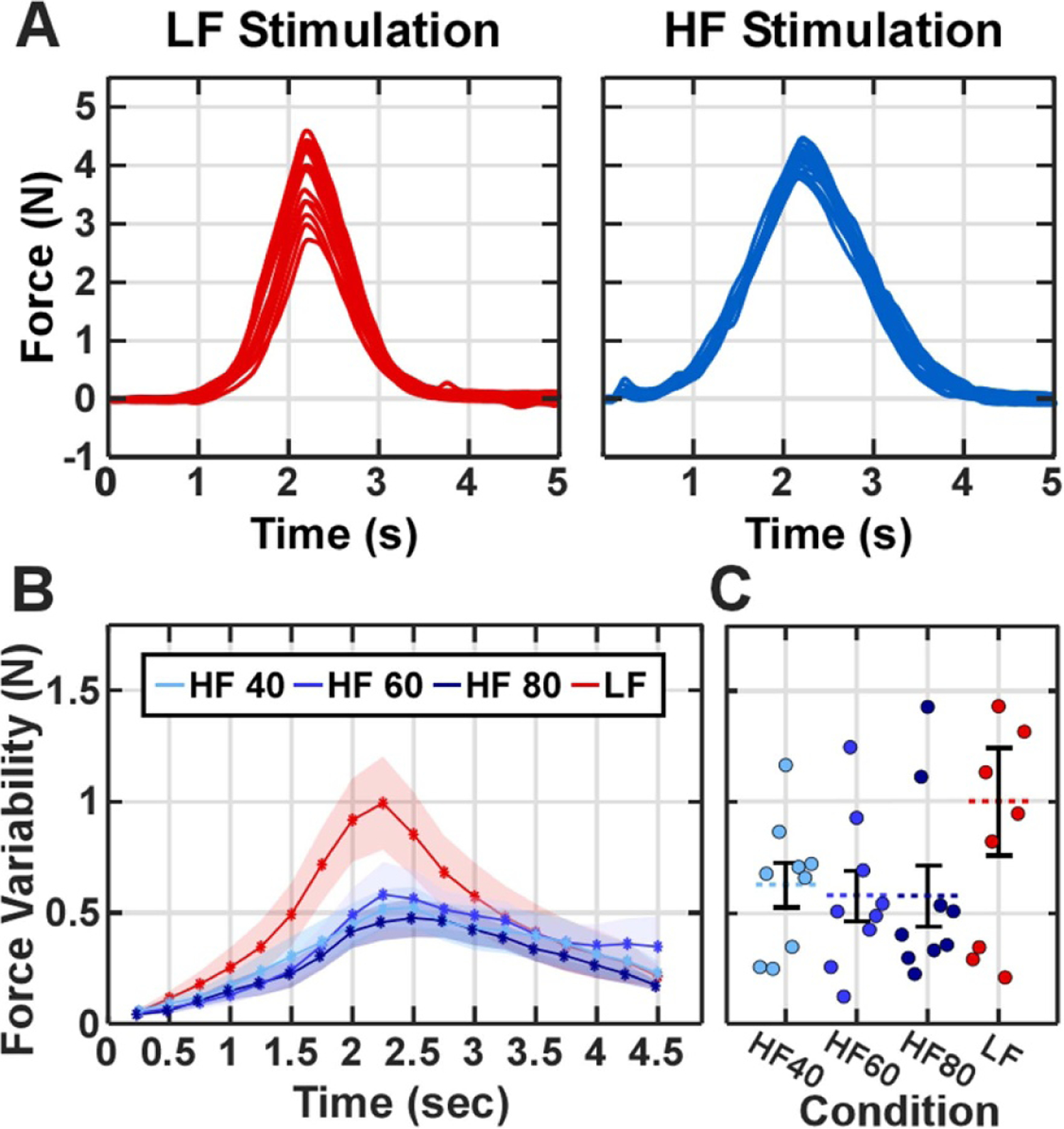
Force variability across pulse repetitions. (A) Force profiles in the low frequency (LF, left) and high frequency (HF, right) stimulation conditions. (B) The trial-to-trial force variability (standard deviation) over time with error bands depicting standard errors across participants. (C) Maximum force variability across participants. The circles in each condition represent the individual subjects ordered from left to right based on the subject number. We used a Kruskal–Wallis test to evaluate significance (*H* = 2.33, *p* = 0.51). One participant during the LF trials exhibited an average force variability of 2.52 N, which is not plotted to maintain clarity but was included in the analysis.

We then quantified the variability of the evoked muscle forces across different stimulation conditions. Figure 5(A) shows the evoked force output profile across all 20 repetitions for a representative LF and HF trial. The force variability (standard deviation) over time (figure 5(B)) was greater during the LF trials than during all three HF conditions near the peak force values. The variability (standard deviation) of the maximum force output (figure 5(C)) was greater during LF trials; however, due to variability across subjects, a significant difference was only noted between HF 80 and LF trials (*p <* 0.05).

### 3.2. Simulation results

**Figure 6.**
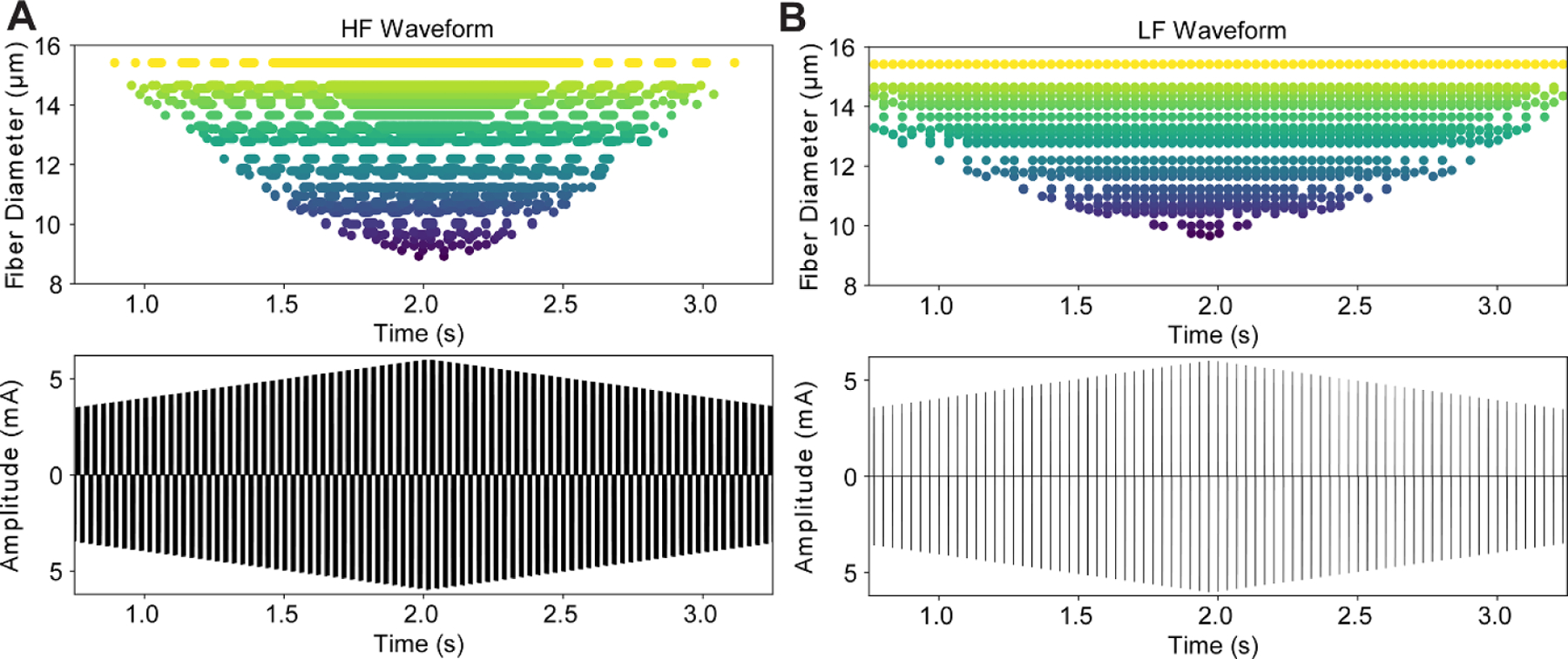
Response of different diameter model mammalian myelinated nerve fibers to ramped amplitude waveforms. (A) High frequency (HF) subthreshold kilohertz waveform. The largest diameter nerve fibers (yellow) were activated at the lowest current amplitudes and the threshold current increased as the fiber diameter decreased (from yellow through green to purple). (B) Rectangular partial duty cycle waveform with 0.5 ms phase^−1^ biphasic pulses at 30 Hz. Again, the largest diameter nerve fibers (yellow) were activated at the lowest current amplitudes and the threshold current increased as the fiber diameter decreases (from yellow through green to purple).

**Figure 7.**
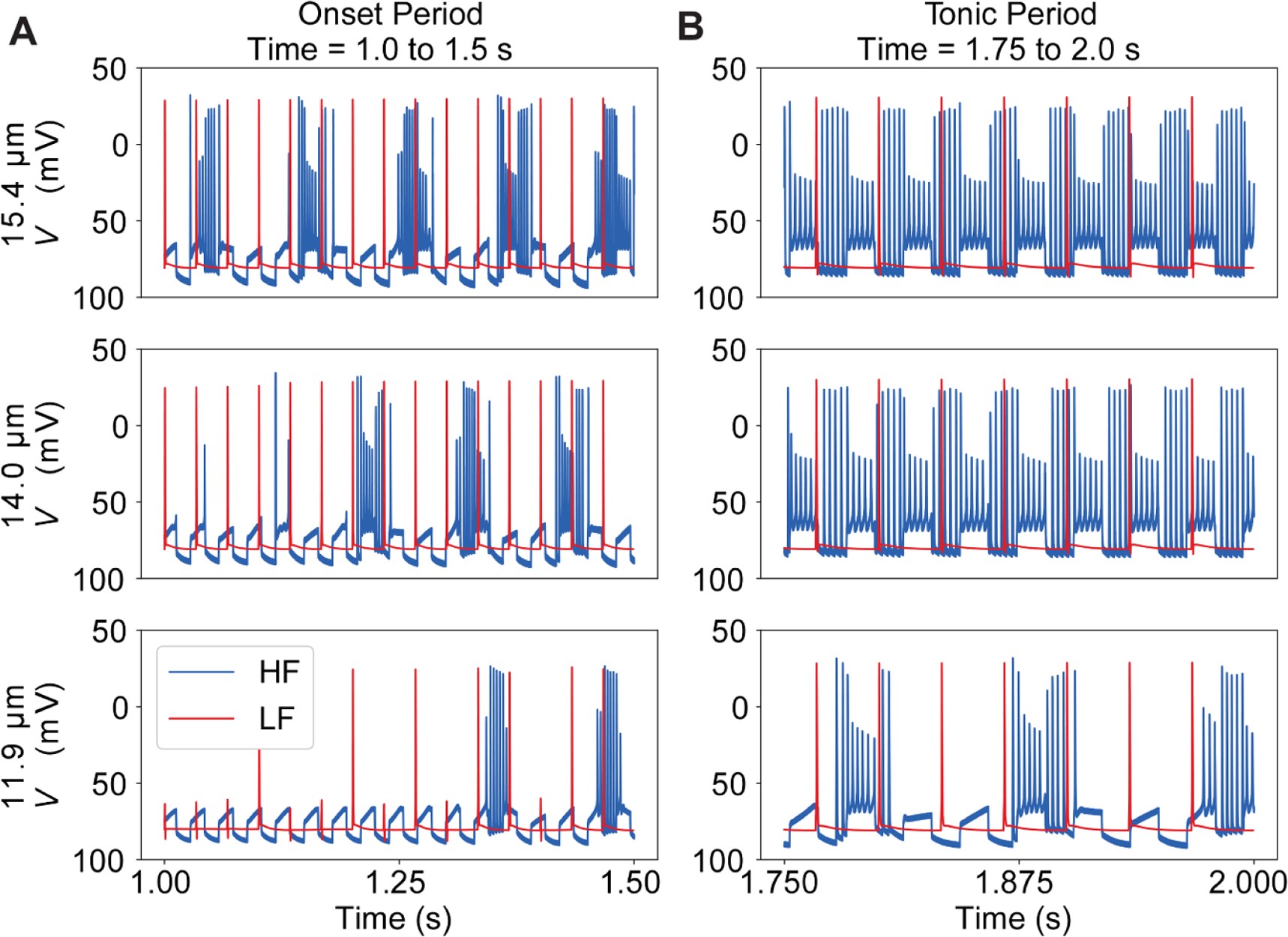
Transmembrane potential beneath one of the point current source stimulating electrodes during the (A) onset and (B) tonic activation periods. Transmembrane potential for three different fiber diameters which are plotted from largest diameter (i.e. lowest threshold and fastest conduction velocity; top row) to lowest (i.e. highest threshold and slowest conduction velocity, bottom row). The fiber responses to the HF (blue) and LF (red) waveforms are plotted on the same axes to show differences in fiber responses. We confirmed that the short action potentials in the tonic period propagated to the end of the fiber.

In response to the HF waveform, the largest diameter fibers were activated at the lowest stimulation amplitudes. With increasing stimulation amplitude, smaller diameter fibers were activated (figure 6(A)). Just above threshold, fibers responded to each burst of stimulation pulses with a single action potential (figure 7(A), see 14 *µ*m fiber). As the amplitude of the waveform increased, fibers responded with bursts of action potentials that lasted longer than the duration of a single burst of pulses followed by periods of quiescence. At even higher amplitudes, fibers responded with tonic activation (figure 7(B)). During burst and tonic responses to HF stimulation, the number of action potentials was much smaller than the number of pulses delivered, suggesting that the membrane was integrating kHz frequency pulses to generate an action potential and that pulses were delivered faster than the fibers’ recovery period. As a result, the activated fibers fired asynchronously in response to HF stimulation. As the amplitude ramped down the sequence reversed (i.e. tonic activation, bursts, then single action potentials) (figure 6(A)).

In response to the ramped LF (30 Hz) symmetric rectangular biphasic pulses (figure 6(B)), fibers were activated at lower stimulation amplitudes than with the HF waveform (figure 6(A)). Again, larger diameter fibers were activated at lower stimulation amplitudes. However, as the amplitude increased beyond threshold, rather than evoking bursting or tonic activity, fibers of all diameters responded to each pulse with a single action potential (i.e. action potentials fired synchronously and entrained to the stimulus).

In summary, with the HF stimulation waveform, action potentials were more asynchronous with respect to the timing of the stimulation bursts and across the distribution of fiber diameters than with the LF waveform. Further, for the HF waveform there were periods when smaller diameter fibers were active and larger diameter fibers were not, while this was not observed with the LF pulse train. Using the LF waveform, fibers of all diameters were activated synchronously with a suprathreshold pulse.

## 4. Discussion

We quantified the responses to a novel kilohertz electrical stimulation waveform with subthreshold current pulses. We hypothesized that axons with different diameters would require different numbers of temporally summed subthreshold depolarizations thus resulting in asynchronous action potentials across axons. Through experimental measurements of EMG and model simulations, we found that HF activated axons in an asynchronous manner. Our simulation results provide direct evidence of asynchronous firing patterns with our HF waveform, while conventional LF stimulation elicited synchronized time-locked responses to the stimulation pulses. Similarly, our experimental results revealed that the EMG activity evoked by HF stimulation shows high entropy (asynchronous) firing activity similar to voluntary EMG activity, rather than the low entropy compound action potentials evoked by conventional LF stimulation. The muscle forces evoked by HF stimulation also showed more consistent force profiles across repeated stimulation trials compared with LF stimulation. Our data indicate that HF stimulation with subthreshold pulses may improve FES with more controllable muscle force output. In summary, this study introduces a novel stimulation waveform that can provide more functional motor control, thereby potentially improving the clinical impact of FES systems for individuals with motor impairment.

### 4.1. Stimulation dependent fiber responses

Our modeling results demonstrate more asynchronous firing of fibers with different diameters in response to HF compared to LF. Asynchronous firing patterns during kHz stimulation has been shown previously in models of deep brain stimulation [19] and vagus nerve stimulation [21]. The mechanism of the asynchronous firing patterns observed in the current study was due to temporal summation of varying numbers of membrane depolarizations. In response to our HF waveform, like our experimental findings, we did not observe transient changes (i.e. initial synchronized firing, and then change to asynchronized firing) as observed in earlier studies [20]. However, as stimulation amplitude increased we observed a transition from single activation to burst activation and then tonic activation. The highly synchronized firing events aligned with the LF stimulation pulses are consistent with our experimental findings that show low entropy compound action potentials with high amplitudes and narrow peaks. A previous study evaluated the performance of HF waveforms that were more similar to conventional LF waveforms [33]; the HF burst duration was 1.5–4 ms, which was more similar to the 0.6–1 ms pulse width of the LF waveform. The asynchronous activation response in the previous study was 1.5–4 ms, which matched the HF burst duration, and we observed distinct compound action potential waveforms. In contrast to the previous study, the continuous HF stimulation used in the current study allowed asynchronous activation of axons throughout the stimulation duration and the degree of asynchronous activation (i.e. EMG entropy) was much higher. The asynchronous activation of axons can lead to better control of the muscle forces during clinical applications with less variability of elicited forces since different force twitches are asynchronous. Previous studies also demonstrated that asynchronous activation of different muscle fibers can delay the onset of muscle fatigue [34–36]. Therefore, the asynchronous axonal activation with our novel HF stimulation waveform could potentially reduce the degree of muscle fatigue for assistive and rehabilitation applications.

For both stimulation waveforms, in our simulations we observed inverse recruitment order of fibers with diameter (i.e. larger diameter axons were recruited at lower stimulation amplitudes than smaller diameter fibers). However, because of the asynchronous patterns of activation, HF stimulation generated periods when the small diameter fibers were active without large diameter fiber activation, while such periods were not observed during the synchronous activation from LF stimulation. It is important to highlight that the model simulation only included direct activation of motor neuron axons and did not consider potential contributions of reflex pathways. In the median nerve bundle segment near the upper arm, over 80% of fibers are sensory fibers [37]. Therefore, at low to moderate stimulation amplitudes, the recruited fibers may include many larger diameter, low threshold sensory fibers, which may activate muscles through reflex pathways. In this case, the spinal motoneurons are recruited in an orderly manner following the size principle [38]. Nevertheless, further investigation is needed to quantify the degree of involvement of reflex pathways.

### 4.2. Evoked EMG

Consistent with the simulation results, we found that evoked EMGs during HF stimulation *activity* exhibited high entropy signals with action potentials occurring at different timings with complex interference patterns, similar to voluntary EMG signals. In contrast, the EMG activity evoked by LF stimulation showed simple-to-identify compound action potentials (i.e. a mix of M-wave and H-reflex responses depending on the stimulation intensity and recording channel [39]) and low entropy values. Asynchronous axonal firing patterns and corresponding motor unit action potentials result in constructive and destructive interference between motor unit action potentials, thereby increasing the entropy of the EMG signal. Since the stimulation pulses at the beginning of each stimulation train were subthreshold, we did not observe transient changes in EMG activity during the initiation of stimulation as observed in previous studies [20]. The individual current pulses in the HF waveform did not stimulate the axons. Rather, the HF waveform generated activation at a range stimulation amplitudes based on the recruitment threshold of different axons, more similarly to how neurons are activated voluntarily [40]. In the current study, we examined three pulse width values (40, 60, and 80 *µ*s) with a fixed pulse interval of 20 *µ*s. We did not observe any difference among these parameter settings in EMG signal entropy. However, we expect that waveforms with longer pulse widths and lower pulse frequencies will likely evoke more synchronous firing activity.

### 4.3. Evoked muscle forces

During both LF and HF stimulation, a single current pulse or a burst of pulses was delivered at 30 Hz. If the axons were recruited synchronously, we expected that the frequency of the evoked force twitches would be 30 Hz. Indeed, power spectra showed that 30 Hz is a dominant frequency for the LF stimulation waveform. In contrast, similar to the voluntary force spectrum, HF stimulation did not evoke much power at 30 Hz, indicating that the evoked force twitches are temporally dispersed across motor units. In addition, our simulation results showed that different fibers activated at different frequencies and different times. Our simulation results also showed that axons can be activated multiple times within one HF cluster. The lowest threshold axons in the nerve are primarily sensory axons [37], which can fire at rates much higher than 30 Hz and could affect motoneuron activity reflexively. However, further investigation is needed to assess the effect of any reflexive contribution to force output and fatigue.

We also quantified force variability across repeated stimulation trials. LF stimulation evoked more variable force profiles across trials than HF stimulation. We observed the most force variability at high force levels. Greater variability of force during LF can be attributed to several factors. First, synchronously superimposed motor unit twitch forces tend to produce more variable forces than temporally dispersed twitch forces [41, 42]. A motor unit force twitch duration can range from 10 to 100 ms, and a faster force twitch is associated with a larger twitch amplitude by a larger motor unit [43]. At 30 Hz stimulation, faster and larger twitches cannot overlap to produce a sustained fusion force [44], and, during synchronous activation, gaps in motor unit force are not smoothed out by twitch forces of other (slower) motor units, thereby resulting in higher variability of evoked muscle force. The HF stimulation waveform produced asynchronous firing which can smooth out the forces from a series of motor unit twitches. Second, different axons and associated muscle fibers were activated synchronously at 30 Hz (except during the initial recruitment and de-recruitment at low amplitudes), as determined by the stimulation frequency. However, during voluntary activation, different motor units are activated at different frequencies determined by their physiological properties and levels of descending input. For example, low and high threshold motor units with different peak firing rates can generate sustained muscle force output [45]. Consistent force output is important for precise control using FES, especially for fine control of hand muscles during activities of daily living. Overall, we observed greater variability during LF trials, but no significant differences were noted across HF pulse widths. The current study evaluated the evoked force variability without voluntary input, and the overall force variability may change when voluntary effort is performed during the stimulation.

### 4.4. Limitations

The current study has several limitations. First, we examined three pulse width values in the HF stimulation waveforms with a fixed 20 *µ*s pulse interval. These parameters were selected based on earlier work [33, 46] and the 20 *µ*s time resolution of our stimulator. Future work is necessary to explore further the temporal parameter space to identify the optimal settings that can lead to more stable and sustainable force profiles. Second, we used simplified models to reduce the computational load, which was a compromise to enable capturing the high temporal resolution of axonal membrane dynamics over several seconds. Nonetheless, the simplified model enabled quantifying axon firing patterns during LF and HF stimulation. Using a realistic physical geometry model (e.g. derived from finite element construction of tissue), we expect a similar firing pattern in the axons at more accurate stimulation amplitudes. In future studies, we plan to construct individual-specific geometric models. Further, our current model does not incorporate contributions of reflex pathways. With the addition of the spinal reflex circuity, we expect activation of sensory fibers at low to moderate stimulation intensity, thereby recruiting motoneurons following the size principle. We selected a moderate force level for the experiment to minimize discomfort and fatigue, which is also relevant for potential clinical applications because lower finger forces are used during most activities of daily living [24]. However, the peak amplitude of stimulation may not recruit the highest threshold small diameter axons. Nonetheless, we found asynchronous axon firing in our experimental and simulation data for fibers of different diameters.

## 5. Conclusions

We presented a novel kHz stimulation waveform that evoked force profiles with less variability and that more closely resembled forces from voluntary contraction than conventional LF stimulation. Our model simulations and experimental measurements both demonstrated that HF stimulation evoked asynchronous neural activation across fibers. The HF evoked activation patterns lead to EMG activity more similar to voluntary activation. These features may provide functional benefit for fine control of muscle forces in individuals with neuromuscular conditions.

## Supplementary Material

supplemental results

## Figures and Tables

**Table 1.** Stimulation electrode pair, amplitude ranges during LF and HF trials, and finger evaluated for each participant.

Subject #	Electrode pair (cathode–anode)	LF stim range (mA)	HF stim range (mA)			Finger evaluated
			HF 40	HF 60	HF 80	
1	13–15	2.8–5	1.6–4	1.6–4	1.6–3.4	Index
2	11–13	1–2.8	0.6–2.5	0.6–2.3	0.5–2.1	Middle
3	12–14	2.5–5.5	1.8–4.6	1.8–4.6	1.5–4.5	Middle
4	5–7	3.5–6	2.6–4.9	2.4–4.6	2.5–4.5	Middle
5	5–12	2.5–5.7	1.6–4.6	1.6–3.8	1.6–4.4	Middle
6	11–13	2.2–4.1	1.6–3.6	1.4–3.0	1.4–3.3	Index
7	10–12	2–4	1.3–2.5	1.2–2.2	1–1.8	Index
8	2–4	2.2–4.8	1–3.4	1–3.0	1–3.0	Middle
9	4–6	1.4–2.9	1–2.2	1–2.0	1–1.7	Middle

## Data Availability

The data cannot be made publicly available upon publication because the cost of preparing, depositing and hosting the data would be prohibitive within the terms of this research project. The data that support the findings of this study are available upon reasonable request from the authors.
