# Supplementary material for: Asynchronous axonal firing patterns evoked via continuous subthreshold kilohertz stimulation
Source: J Neural Eng. Author manuscript; Available in PMC 2023 Aug 17. (PMC10433012; doi:10.1088/1741-2552/acc20f)
Supplement: supplemental results [file NIHMS1919377-supplement-supplemental_results.pdf]

Supplementary Material

Asynchronous Axonal Firing Patterns Evoked via Continuous Subthreshold Kilohertz Stimulation

Luis Vargas, Eric D. Musselman, Warren M. Grill, Xiaogang Hu

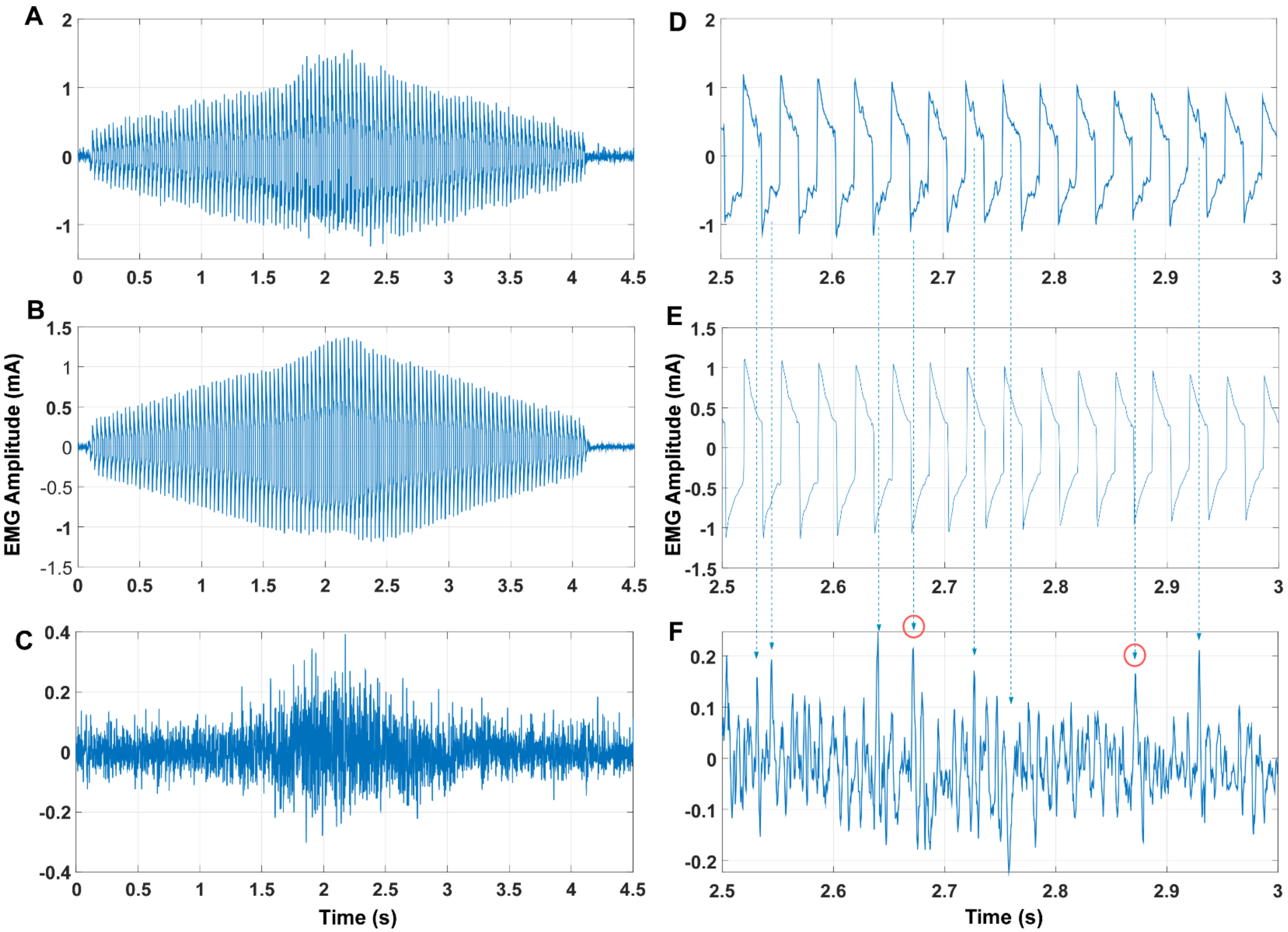

Figure S1: Methods for EMG stimulation artifact removal. **A**: Raw EMG signal with stimulation artifact for one channel. **B**: Estimated stimulation artifact of the same channel. **C**: EMG signal after stimulation artifact removal. We subtracted panel **B** from **A** to get **C**, and before the subtraction the signal in panel **B** was shifted left and right as well as scaled up and down to find the best match (minimum mean-squared-error) with **A**. Panel **D**, **E**, and **F**: corresponding signals of **A**, **B**, and **C** in a zoomed time window. The vertical dotted arrows indicate locations of action potentials from **D** to **F**. The arrows with red circles indicate potential errors from the artifact removal procedure.

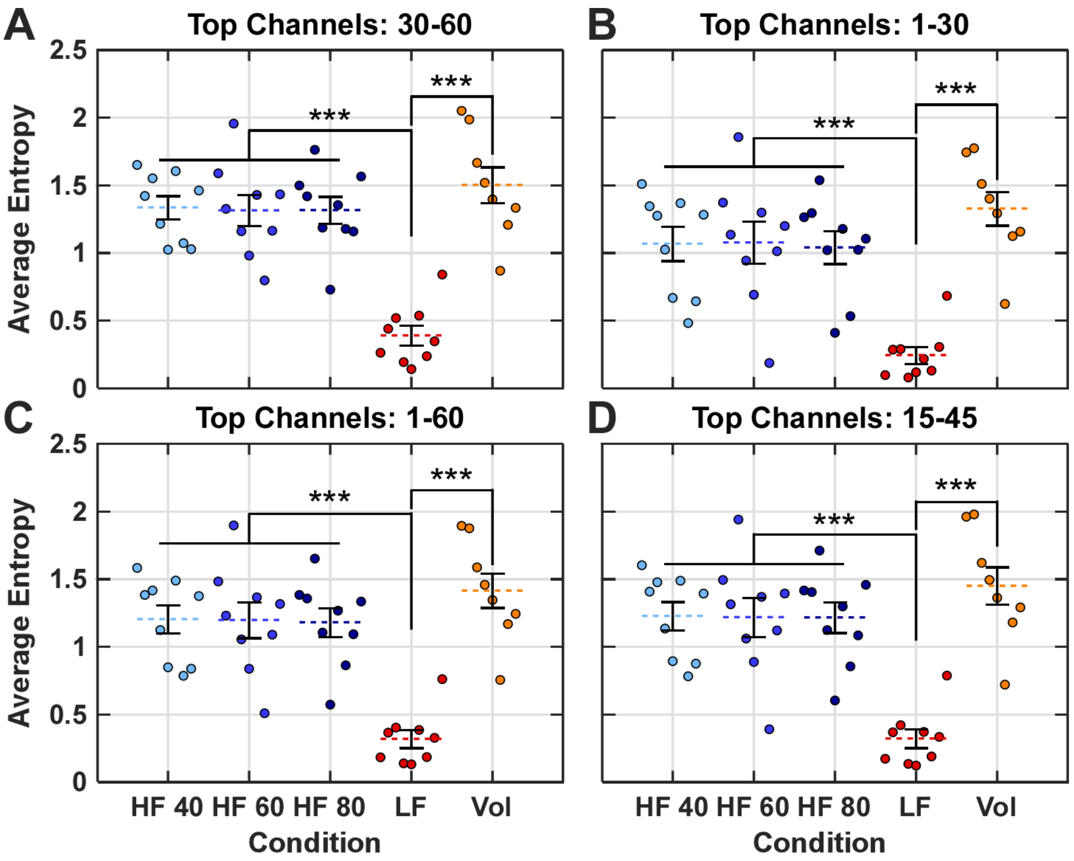

Figure S2. Sensitivity of EMG entropy analysis to EMG channel selection. We assessed three high frequency (HF) conditions, labeled as "HF 40" denoting HF stimulation with a 40 µs pulse width, "HF 60" denoting HF stimulation with a 60 µs pulse width, and "HF 80" denoting HF stimulation with an 80 µs pulse width. We organized the 128 channels across the EMG grid in a descending order based on RMS value. We calculated sample entropy for each waveform using various channel ranges, including **A:** 30th to 60th top channels, **B:** 1st to 30th top channels, **C:** 1st to 60th top channels, and **D:** 15th to 45th top channels. For each panel, each participant is represented as an individual circle and the error bars represent standard errors across all participants. We used a one-way ANOVA to evaluate significance (**A**: $F=18.7$, $p<0.001$; **B**: $F=11.3$, $p<0.001$; **C**: $F=15.1$, $p<0.001$; **D**: $F=14.0$, $p<0.001$) followed by a Tukey's honestly significant difference (HSD) test for post hoc paired comparisons. *** denotes $p<0.001$
